# Preventing carbon nanoparticle-induced lung inflammation reduces antigen-specific sensitization and subsequent allergic reactions in a mouse model

**DOI:** 10.1186/s12989-015-0093-5

**Published:** 2015-07-04

**Authors:** Matthias Kroker, Ulrich Sydlik, Andrea Autengruber, Christian Cavelius, Heike Weighardt, Annette Kraegeloh, Klaus Unfried

**Affiliations:** IUF – Leibniz Institut für Umweltmedizinische Forschung, Auf’m Hennekamp 50, 40225 Düsseldorf, Germany; INM – Leibniz Institut für Neue Materialien, Saarbrücken, Germany; LIMES Institut, Universität Bonn, Bonn, Germany

**Keywords:** Asthma, Environmental air pollution, Ultrafine particles, Compatible solutes, Ectoine, Pulmonary inflammation

## Abstract

**Background:**

Exposure of the airways to carbonaceous nanoparticles can contribute to the development of immune diseases both via the aggravation of the allergic immune response in sensitized individuals and by adjuvant mechanisms during the sensitization against allergens. The cellular and molecular mechanisms involved in these adverse pathways are not completely understood. We recently described that the reduction of carbon nanoparticle-induced lung inflammation by the application of the compatible solute ectoine reduced the aggravation of the allergic response in an animal system. In the current study we investigated the influence of carbon nanoparticles on the sensitization of animals to ovalbumin via the airways. Ectoine was used as a preventive strategy against nanoparticle-induced neutrophilic lung inflammation.

**Methods:**

Balb/c mice were repetitively exposed to the antigen ovalbumin after induction of airway inflammation by carbon nanoparticles, either in the presence or in the absence of ectoine. Allergic sensitization was monitored by measurement of immunoglobulin levels and immune responses in lung and lung draining lymph nodes after challenge. Furthermore the role of dendritic cells in the effect of carbon nanoparticles was studied *in vivo* in the lymph nodes but also *in vitro* using bone marrow derived dendritic cells.

**Results:**

Animals exposed to antigen in the presence of carbon nanoparticles showed increased effects with respect to ovalbumin sensitization, to the allergic airway inflammation after challenge, and to the specific T_H_2 response in the lymph nodes. The presence of ectoine during the sensitization significantly reduced these parameters. The number of antigen-loaded dendritic cells in the draining lymph nodes was identified as a possible cause for the adjuvant effect of the nanoparticles. *In vitro* assays indicate that the direct interaction of the particles with dendritic cells is not able to trigger CCR7 expression, while this endpoint is achieved by lung lavage fluid from nanoparticle-exposed animals.

**Conclusions:**

Using the intervention strategy of applying ectoine into the airways of animals we were able to demonstrate the relevance of neutrophilic lung inflammation for the adjuvant effect of carbon nanoparticles on allergic sensitization.

**Electronic supplementary material:**

The online version of this article (doi:10.1186/s12989-015-0093-5) contains supplementary material, which is available to authorized users.

## Background

Epidemiological studies demonstrated correlations between exposure to particulate air pollution and the incidence and severity of allergic diseases of the airways [1]. Besides sources like environmental tobacco smoke, ultrafine particles from traffic related air pollution have been suggested as modulators of allergic reactions leading to adverse health effects [2, 3]. With the increasing use of nanomaterials in daily life products, human airways might be exposed to inhalable poorly soluble nanoparticles including combustion derived carbon nanoparticles from toners and printers [4]. Carbonaceous nanoparticles in the airways can contribute to both, the exacerbation of the immune response in antigen-exposed sensitized individuals, but also the allergic sensitization against daily life antigens [5, 6].

Mechanistic animal studies demonstrated that the exposure to different kinds of allergens in the presence of diesel exhaust particles (DEP) leads to a potentiation of the antigen-specific sensitization [7, 8]. Comparing chemically different kinds of diesel samples revealed that the amount of organic carbon compounds contaminating the elementary carbonaceous core significantly modulates the strength of this adjuvant effect on the sensitization against antigens [9]. A number of studies however also demonstrate that ultrafine engineered carbon nanoparticles (CNP) which contain only traces of organic carbon enhance the sensitization [10]. In these studies, the increase of antigen-specific IgE was associated with lung inflammation induced by CNP when applied to the airways of the animals [11]. The comparison of chemically similar particle samples of different size classes demonstrate an increasing effectivity associated with the decrease in primary particle size [12, 13]. The influence of spark-generated carbon nanoparticles on immune reactions of the airways has been shown at the level of the immune response after challenging sensitized mice [14]. In a recent study this exacerbating effect was investigated in asthmatic volunteers who were exposed to CNP or filtered air in a cross over design prior to allergen challenge. Interestingly, a delayed effect of CNP on the strength of allergic airway inflammation was observed [15]. In a murine model, the occurrence of markers of oxidative stress was associated with the exacerbation of the allergic lung inflammation by the inhaled CNP [16]. The enhancement of the antioxidant capacity of the airways by adding N-acetyl cysteine led to a significant reduction of the exacerbating effect of the inhaled CNP. Taken together, these studies indicate that the enhanced inflammatory response of the airways as well as the oxidative burst in response to the inhalation of ultrafine particles might be a central mechanism of adverse effects of inhaled nanoparticles on immune diseases of the airways. Strategies which reduce airway inflammation should therefore be able to prevent both the exacerbation of the immune response in sensitized individuals but also the adjuvant effect during allergic sensitization.

We recently described the potential of a group of substances, named compatible solutes, to prevent CNP-induced pro-inflammatory reactions in the airways of exposed animals [17]. These substances can be isolated from extremophilic bacteria which produce them in order to stabilize cellular functions under extreme life conditions like heat or osmotic stress. Based on the principle of preferential exclusion, compatible solutes stabilize macromolecules like membrane lipids and proteins [18–20]. In lung epithelial cells, we were able to demonstrate that CNP-triggered non-canonical activation of EGFR signalling is prevented in the presence of ectoine due to a stabilization of the receptor in the lipid raft signalling compartment of the cells [21]. Addressing inflammatory kinetics in the airways *in vivo*, we observed similar effects of CNP and of ectoine on cell stress reactions executed on neutrophilic granulocytes (PMN). CNP contribute to the aggravation of the inflammation by suppressing natural apoptosis rates via membrane dependent signalling in both human peripheral PMN and *in vivo* in exposed rat lungs [22]. The preventive application of ectoine restored apoptosis rates and led to an accelerated resolution of the neutrophilic lung inflammation. As a proof of the biophysical principle of preferential exclusion, we observed that besides ectoine which is isolated from halophilic bacteria, firoin a substance produced by thermophilic bacteria, is also able to prevent the adverse cell stress reactions in lung epithelium and in neutrophils [23].

The possibility to prevent CNP-induced neutrophilic lung inflammation directly at the level of PMNs offers the possibility to test whether this early reaction of the airways contributes to the development of responses of the adaptive immune system after allergen challenge. In a recent report we described that the aggravation which is mediated by CNP applied during antigen challenge in sensitized mice can be significantly reduced in the presence of ectoine [24]. The current study aimed to investigate how the adjuvant effect of CNP during sensitization against ovalbumin is mediated and whether it is reduced when neutrophilic lung inflammation is attenuated by the application of the compatible solute ectoine. For that purpose, we chose the experimental system of pharyngeal aspiration in which the strength of neutrophilic lung inflammation can be strictly controlled by ectoine. Sensitization via the lower airways and adaptive immune responses after sensitization and challenge were investigated using a mouse model of allergic airway disease in Balb/c mice. In order to separate effects of neutrophilic lung inflammation from direct effects of particles and ectoine on antigen presenting cells, mechanistic studies employing *ex vivo* differentiated dendritic cells were performed.

## Results and discussion

As a pre-requisite for *in vivo* and *in vitro* investigations with CNP, particle suspensions were analysed for their physico chemical characteristics. CNP (14 nm, primary diameter) are known to form agglomerates in physiological aqueous solution. Characteristics of the particle suspensions are given in the supplementary data (Additional file 1: Figure S1 and Table S1). In earlier studies we applied such agglomerated nanoparticles *in vitro* and *in vivo* and observed size or surface specific effects when compared to bigger non-nano particles which showed very similar agglomerate size distributions [21].

**Fig. 1:**
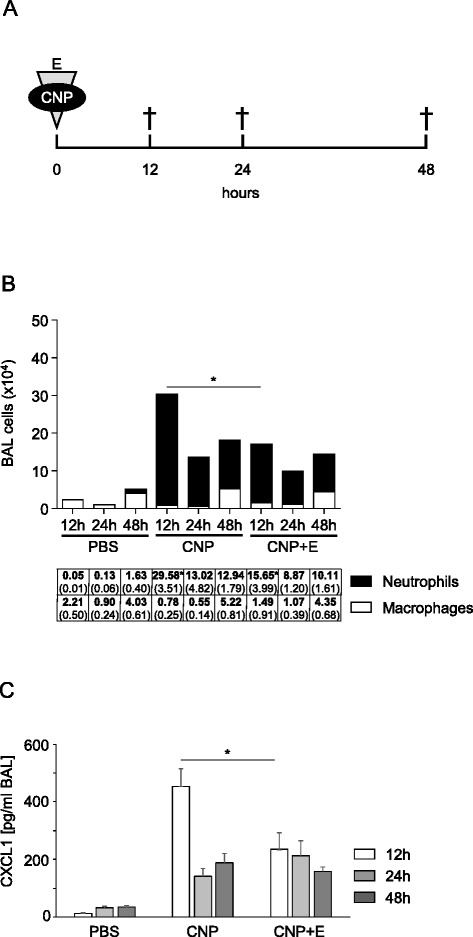
Ectoine application reduces neutrophilic lung inflammation induced by CNP in Balb/c mice. **a** Experimental design, animals (*n* = 5) were exposed to PBS (control), 2.5 mg/kg CNP, or 2.5 mg/kg CNP with 1 mM ectoine (E) and subsequently sacrificed at the indicate time points. **b** Differential cell numbers in BAL (means, SEM). **c** CXCL1 levels in BAL. * significant differences were observed in total cell numbers, neutrophil numbers and CXCL1 levels (*p* < 0.05, Mann Whitney *U*-test)

In a pilot animal study, the effect of ectoine on the lung inflammation induced by the pharyngeal aspiration of 2.5 mg/kg CNP was investigated after 12, 24, and 48 h (Fig. 1a). A mild inflammatory increase in total cells numbers and specifically in neutrophils was observed 12 h after single application of CNP in the broncho-alveolar lavage (BAL) as compared to BAL of control mice (Fig. 1b). Following the kinetics of the lung inflammation, at later time points decreasing numbers of neutrophils were observed, while the number of clearing macrophages was increased. Accordingly, the application of 1 mM ectoine together with the particles led to a reduction of total cell numbers due to the significantly lower neutrophil numbers. Ectoine had no significant effect on the number of macrophages at all time points. This effect was nicely reflected at the level of the neutrophil recruiting chemokine CXCL1 (homologue to human IL-8). In earlier studies we demonstrated that the effect on the reduction of neutrophilic lung inflammation is not due to changes in particle properties but is caused by the effect of ectoine on membrane dependent signalling processes both in lung epithelial cells and in neutrophils [17]. As previously published, ectoine also reduced the amount of cytokines like IL-5 and IL-6 in BAL of exposed mice after 12 h [24]. The data demonstrate that neutrophilic lung inflammation induced by pharyngeal aspiration of CNP can be modulated by the application of ectoine. This system enabled us to study the influence of the inflammatory reaction on allergic sensitization via the lower airways.

**Fig. 2:**
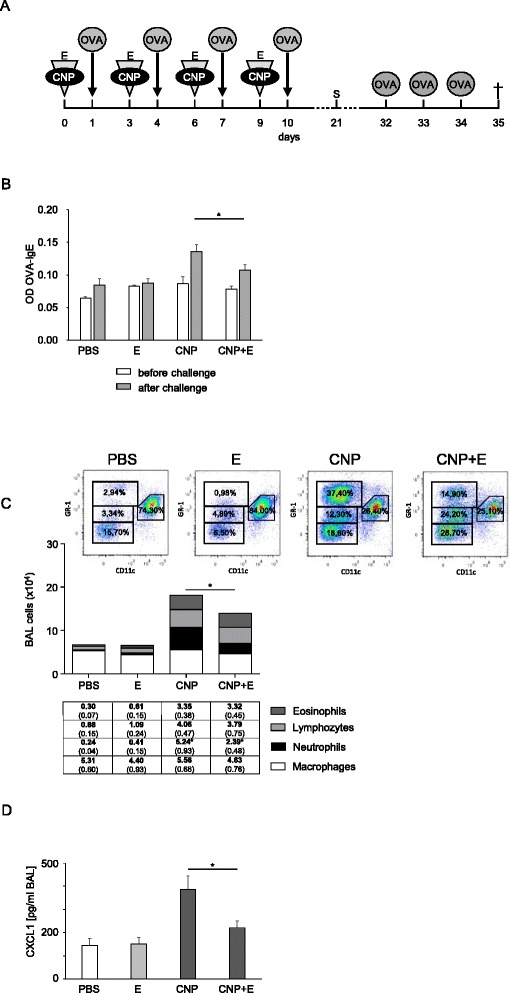
CNP exposure during sensitization leads to enhanced immune responses which can be attenuated by ectoine. **a** Experimental design, for sensitization, animals (*n* = 8) were treated with PBS, CNP, and CNP + E as described in Fig. 1. Additionally, 12 h after this treatment animals received 20 μg OVA. At day 21 serum was collected (S). After challenge (1 % OVA in PBS, 30 min) on three consecutive days (d32 – d34), animals were sacrificed and BAL, blood and lymph nodes were collected. **b** OVA-specific IgE prior to and after challenge. **c** Differential cell counts (representative plots and means, SEM) after challenge significance bar indicates differences in total cell numbers and neutrophil numbers. **d** CXCL1 levels in BAL after challenge. *p < 0.05, Mann Whitney *U*-test

Therefore, the effect of CNP on allergic sensitization was tested in a model of allergic airway inflammation *in vivo* (Fig. 2a). Lung inflammation was induced by the application of 2.5 mg/kg CNP in the presence or absence of 1 mM ectoine. At the peak of the inflammatory response, after 12 h, OVA was applied to the lower airways also by pharyngeal aspiration. After a recovery period of two days each, the procedure was repeated three times. In order to induce an allergic immune response, animals were challenged in an exposure chamber on three consecutive days. As described by de Haar et al. [12] sensitization via the airways in Balb/c mice leads to increased allergen specific IgE levels. We therefore monitored OVA specific IgE levels in the serum of the animals pre and post challenge (Fig. 2b). While only non-significant trends in OVA specific immunoglobulin were observed prior to the challenge, a significant increase of this parameter in CNP-exposed animals was observed after boosting. This effect was significantly lower when ectoine was applied to the airways during the sensitization phase, indicating a preventive effect of ectoine on the adjuvant effect of CNP. Similar effects were observed at the level of antigen-induced lung inflammation after challenge (Fig. 2c). BAL cells were identified by flow cytometry after staining with GR-1 and CD11c as described elsewhere [11]. OVA inhalation increased BAL cell numbers only in animals which were sensitized in the presence of CNP while animals sensitized in the presence of CNP and ectoine had significantly less inflammatory cells in the lung. This attenuation is mainly due to the significant reduction of neutrophils which is again reflected by the CXCL1 levels in BAL (Fig. 2d). Interestingly, predominantly the neutrophilic inflammation is affected by ectoine, the enhanced amount of eosinophils and lymphocytes in BAL, which might be induced by the induction of the allergic reaction is rather unaltered. As the inflammatory response is still dominated by neutrophil numbers, the effect on the other cell types might not be visible in the statistical analyses.

**Fig. 3:**
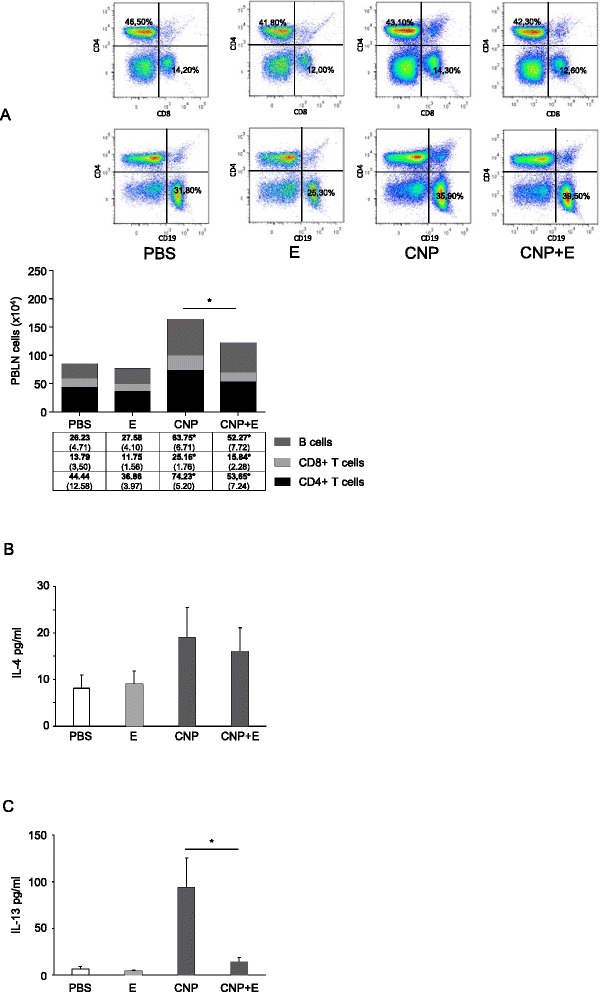
Changes in lymph node responses after challenge. Lymph nodes from the animals (Fig. 2) were analysed for adaptive immune responses. **a** Total cell numbers in peribronchial lymph nodes (representative plots and means, SEM). **b** IL-4 release in re-stimulated lymph node cells. **c** IL-13 release in re-stimulated lymph node cells. *p < 0.05, Mann Whitney *U*-test

In order to verify the effect of CNP on the sensitization against OVA and to further investigate the possible effects of ectoine on this process, we studied the adaptive immune reactions in the peribronchial lymph nodes of the animals at day 35 by flow cytometry (Fig. 3a). Total cell numbers appeared to reflect the sensitization status of the animals. A significant increase in all tested cell types (B cells, CD8^+^ T cells, and CD4^+^ T cells) was observed in animals which were sensitized in the presence of CNP. Numbers of all cell types were lower in the animals in which ectoine was present in the initial experimental phase. As an additional readout for differences in the immune status of the animals, cultured lymph node cells were re-stimulated with OVA and the T_H_2 cytokines IL-4 (Fig. 3b) and IL-13 (Fig. 3c) were determined in the supernatants. Both cytokines were significantly elevated only in cell cultures from animals sensitized in the presence of CNP. A statistically significant reduction was observed for the IL-13 release in the samples from CNP plus ectoine animals, whereas IL-4 levels were not altered significantly by ectoine.

**Fig. 4:**
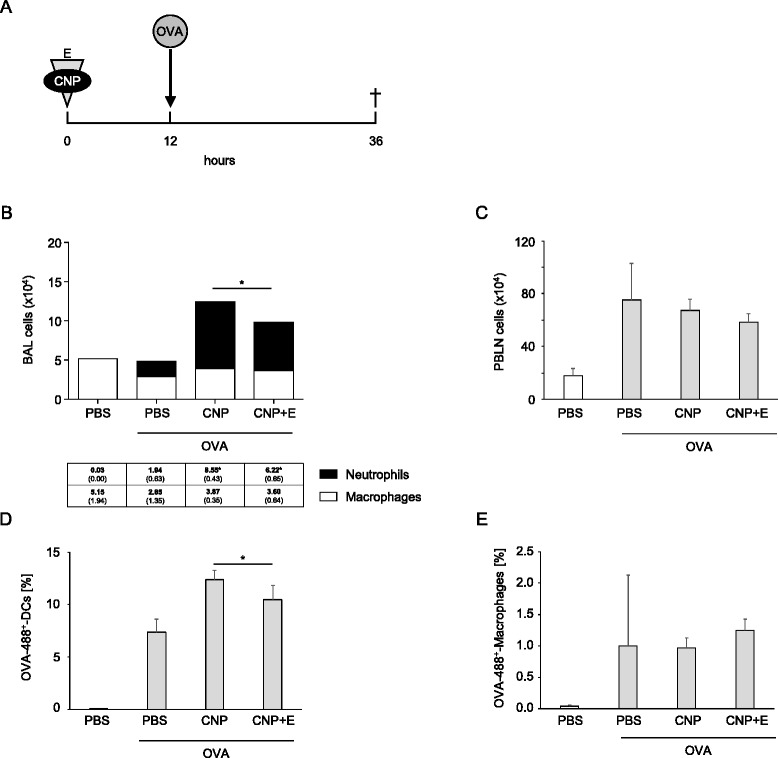
Ectoine application reduces the frequency of antigen loaded dendritic cells in lymph nodes. **a** Animals (*n* = 3 in control groups, *n* = 8 in exposure groups) were exposed to 50 μg Alexa Fluor 488-labelled OVA 12 h after the application of PBS (control), CNP, or CNP + E. In order to control for effects of labelled OVA, an additional PBS group without OVA was employed. **b** BAL cell numbers (means, SEM) 36 h after initial treatment. **c** Total lymph node cells. **d** Percentage of OVA-488 positive dendritic cells (MHCII^+^, CD11c^+^). **e**. Percentage of OVA-488 positive macrophages (MHCII^+^, F4/80^+^). *significant differences were observed in total cell numbers, neutrophil numbers, and percentage of OVA-488 positive dendritic cells (*p* < 0.05, Mann Whitney *U*-test)

The data presented so far demonstrate a strong adjuvant effect of CNP on the process of sensitization against the allergen OVA via the airways. The reduction of the neutrophilic lung inflammation by ectoine application during sensitization suggests that this adjuvant effect is driven by the lung inflammation. Since the immune response in the lung draining lymph nodes is also affected by the exposure to CNP, it could be possible that CNP also directly interact with antigen presenting cells and trigger an immune response besides their capacity to induce a neutrophilic inflammation in the lung. This hypothesis is supported by the finding that CD4^+^ cell proliferation is enhanced in peribronchial lymph nodes after CNP application to the airways in a concentration which appeared not to induce accelerated neutrophil numbers in BAL [25]. Additional data of the same study suggest that the number of antigen presenting dendritic cells is increased by the particle application. We therefore aimed to investigate the role of antigen presenting cells after CNP and ectoine application. In an additional animal experiment, the effects of CNP and ectoine on macrophages and dendritic cells, which are identified as the cell types which transport antigen from the airways to the lymph nodes [26, 27], were investigated (Fig. 4). At the peak of neutrophilic inflammation the fluorescently-labelled antigen (OVA-Alexa Fluor 488) was applied to the airways via pharyngeal aspiration in order to track migrating cells from the lung to the lymph nodes. In order to control for effects of labelled OVA, an additional PBS group without OVA was employed. After 36 h, lung inflammation was determined in BAL and peribronchial lymph nodes were analysed for the presence of antigen carrying cells. Also under these experimental conditions CNP led to an enhanced accumulation of neutrophils in the BAL, while there was an attenuated neutrophil influx after ectoine application (Fig. 4b). This effect was not observed at the level of total lymph node cell numbers (Fig. 4c). Among the dendritic cells (MHCII^+^/CD11c^+^) present in the lymph node, an increased number of OVA-488^+^ cells was observed when the antigen was applied during an ongoing CNP-triggered inflammation (Fig. 4d). This effect was significantly reduced in the presence of ectoine. Only a very low percentage of macrophages (MHCII^+^/F4/80^+^) was shown to be OVA-488 positive and no effect of the ectoine treatment was observed for this cell type (Fig. 4e). Thus, CNP seem to enhance the migration of antigen loaded dendritic cells to the draining lymph nodes and ectoine appears to prevent this effect either via direct action on dendritic cells or via the suppression of the neutrophilic inflammation.

**Fig. 5:**
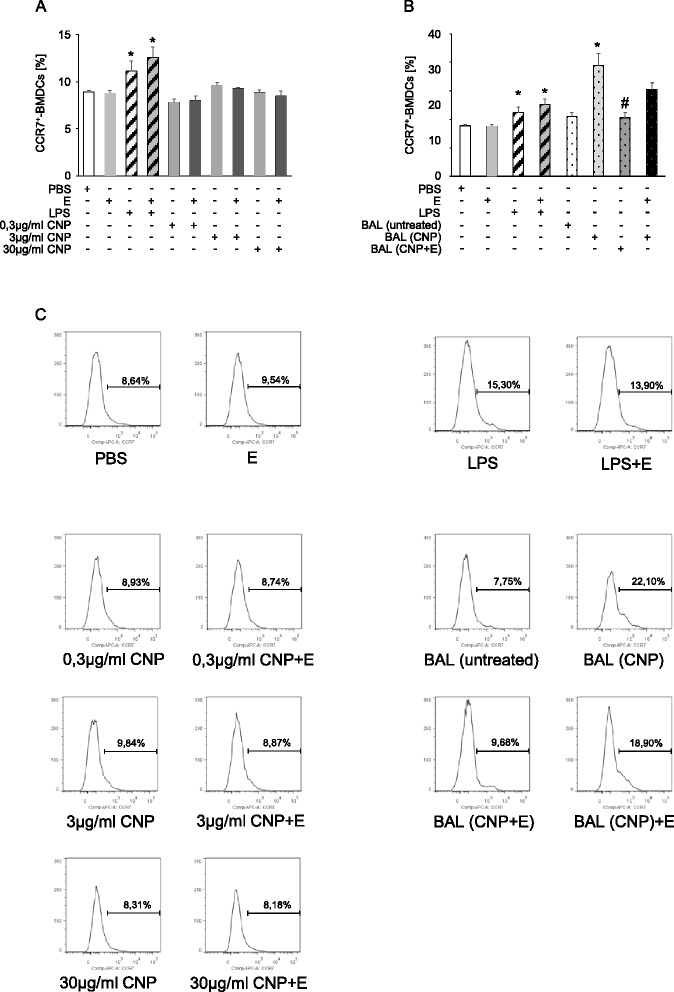
Effects of CNP and lavage fluid on bone marrow derived dendritic cells. Dendritic cells derived from Balb/c mice (*n* = 7) were exposed to the indicated doses of CNP (**a**) or lavage fluid (**b**) from exposed animals. **c** Representative histograms determining CCR7^+^ cells. *significantly different from PBS alone, # significantly different from cells treated with BAL from CNP-exposed animals. (*p* < 0.05, ANOVA with post hoc testing)

To address the question whether these effects are mediated by the particles themselves or rather by factors released during airway inflammation, bone marrow derived dendritic cells were exposed *in vitro* to CNP and to cell free BAL fluids from exposed and control animals (Fig. 5). The cells were tested for the expression of the chemokine receptor CCR7 which is crucial for migration of antigen presenting cells from the periphery to draining lymph nodes [28]. As expected, lipopolysaccharide (LPS) as a positive control enhanced the number of CCR7 positive dendritic cells. This effect was not influenced in the presence of ectoine. Incubating the cells with different doses of CNP suspensions did not lead to an increase of CCR7 positive cells (Fig. 5a). At least under these conditions the nanoparticles had no effect on the dendritic cells. However, treating the cells with lavage fluids, devoid of cells and particles, from animals which had been exposed to CNP for 12 h revealed very clear results (Fig. 5b). A significant increase in CCR7 positive cells was observed after treatment with the lavage from CNP alone treated animals. This response was significantly lower in samples incubated with lavage from CNP plus ectoine-treated animals. The induction of CCR7, however, was not significantly reduced when ectoine was added to the dendritic cell cultures *in vitro* as an intervention strategy against the effects of lavage fluid from CNP-alone animals. Other studies have shown that CNP in contrast to ambient particles do not induce the activation of dendritic cells [29]. Similar results were obtained when we tested bone marrow derived dendritic cells for the expression of CD86 after treatment with CNP and lavage fluid (data not shown).

## Conclusions

Taken together, the data of this study demonstrate the importance of enhanced neutrophilic lung inflammation induced by CNP during allergic sensitization as a promoter of allergy. This is demonstrated at the level of antigen-specific IgE, challenge-induced lung inflammation, and T_H_2 specific changes in the draining lymph nodes. As a possible cause for these effects, the enhanced migration rate of antigen presenting dendritic cells to the lymph node in response to the ongoing inflammation is indicated. The intervention approach using ectoine which moderately but specifically prevents neutrophilic lung inflammation induced by CNP demonstrates the causal connection of particle-induced inflammation and the aggravation of the allergic reaction in this model. This approach, therefore, also offers the possibility to interfere with environmentally-induced immune diseases of the airways like asthma. Ectoine and other compatible solutes have been tested to be highly compatible to mammals. First human studies describing the feasibility and efficacy of ectoine application in upper airways and on the skin suggest to test such compounds as a preventive strategy against adverse health effects in the airways induced by air pollution [30, 31]. Such a strategy might be of relevance for pre-disposed individuals particularly in situations in which exposure of the airways cannot be avoided. This approach is not only relevant for the prevention of environmentally induced effects on the immune system, since engineered nanoparticles have also been described to aggravate airway responses in experimentally induced asthma [32, 33]. Together with the finding that ectoine also reduces the particle-dependent aggravation of the allergic immune reaction, the current study suggests to test this substance in humans to prevent particle induced adverse effects on adaptive immune reactions of the airways.

## Methods

### Particle suspensions

Carbon nanoparticles (CNP, Printex 90) were obtained from Degussa (Essen, Germany). Stock suspensions (1 mg/ml) of particles were prepared in phosphate buffered saline (PBS) as vehicle by sonication for 60 min. Particles and particle suspensions were characterized as described in the supplementary.

### Application via pharyngeal aspiration and bronchoalveolar lavage

Female Balb/cJRj mice (8 weeks old, Janvier, France) were treated with particle suspensions (2.5 mg/kg bodyweight) with or without 1 mM ectoine (Sigma-Aldrich Chemie, Deisenhofen, Germany) or PBS as control solution via pharyngeal aspiration with a volume of 50 μl, under inhalation anaesthesia (isoflurane, 5 % in synthetic air, 2 min). Animals were sensitized by repetitive pharyngeal aspiration with a volume of 50 μl of 1 mg/ml Grade VI Ovalbumin (OVA) (Sigma-Aldrich Chemie, Deisenhofen, Germany) dissolved in PBS. At the indicated time points, mice were challenged by aerosol inhalation (1 % OVA in PBS) for 30 min using a Pari-Boy Nebuliser (Pari, Starnberg, Germany). For labelling of antigen transporting cells, 50 μl of 1 mg/ml Ovalbumin, Alexa Fluor 488 conjugate (OVA-488) (Invitrogen, Carlsbad, CA, USA) was applied via pharyngeal aspiration. Animals were sacrificed by exsanguinations under deep anaesthesia after the indicated exposure times. Bronchoalveolar lavage (BAL) was taken using 4 × 1 ml PBS. The determination of inflammatory cells in BAL by flow cytometry was performed according to the method described by de Haar and colleagues, staining for GR-1 and CD11c [11]. The validity of this method was verified by additional staining procedures for neutrophils (GR-1, CD11b) and macrophages (F4/80, CD11b), for selected samples. All animal experiments were performed after relevant permission according to German animal protection laws.

### Serum collection

Blood was drawn from mice on days 21 and 35 by facial vein puncture. Serum was stored at −80 °C until measurement of OVA-specific IgE antibodies by ELISA (MD bioproducts, St Paul, MN) according to the manufacturer’s instructions.

### Lavage parameters

BAL-cells were isolated and stained for flow cytometry. Cell free lavage fluids were subjected to solid-phase ELISA in order to determine CXCL1, IL-4 and IL-13 (R&D systems, Minneapolis, MN) according to the manufacturer’s instructions.

### Peribronchial lymph node analysis

Peribronchial lymph nodes were isolated immediately after BAL. After 15 min digestion with 1 mg/ml Collagenase D and 300 U/ml DNase I (both Roche, Mannheim, Germany) in PBS at 37 °C single-cell suspensions were made by using a 100 μm cell strainer (BD Bioscience, Franklyn Lake, NJ, USA). Cell suspensions were incubated for 10 min with red blood cell lysis buffer at 4 C. Then cells were counted and stained for flow cytometry using specific antibodies. Cell suspension were cultured in RPMI-1640 medium with 10 % FCS, 1 % L-Glutamine 2 % Pen/Strep (all obtained from Sigma Aldrich) in round bottom 96-well plate (2 × 10^5^ cells per animal/well) and re-stimulated with 100 mg/ml OVA for 4 days at 37 °C, 5 % CO_2_. Levels of IL-4 and IL-13 in culture supernatants were measured by ELISA (R&D systems, Minneapolis, MN) according to the manufacturer’s protocol.

### Flow cytometry

Flow cytometry was performed with a FACScanto II Flow Cytometer (BD Bioscience, BD Bioscience, Franklyn Lake, NJ, USA) and analysed with FlowJo 7.6.5. Fluorescently labelled CD11c (N418), GR-1 (RB6-8C5), MHCII (M5/144.15.2), CD4 (GK1.5), CD8b (H35-17.2), CD19 (MB19-1), CCR7 (4B12), F4/80 (BM8) and biotin anti-APC (APC003) were used in various combinations. Antibodies used were from BioLegend (San Diego, CA, USA), except for CD19 (eBioscience, San Diego, CA, USA), CD4 and CD8 (BD Pharmingen). Dendritic cells and macrophages loaded with OVA-488 were also detected by using flow cytometry.

### *In vitro* bone marrow-derived dendritic cell exposures

Bone marrow–derived dendritic cells (BMDCs) were cultured from bone marrow by using recombinant murine granulocyte-macrophage colony-stimulating factor (rGM-CSF) (PreproTech, Hamburg, Germany). On day 6, cells were harvested and cell purity was analysed by flow cytometry. Cells had an average purity of 70 %. A stock solution/suspension of CNP or lipopolysaccharides (LPS, Escherichia coli 0111:B4; Sigma-Aldrich) was prepared in PBS. Cells were exposed then for 18 h to CNP (0, 3; 3; 30 mg/ml) with or without 1 mM ectoine, LPS (100 ng/ml) with or without 1 mM ectoine, PBS or ectoine alone, cell free BAL obtained from animals which were treated 12 h with CNP alone or in combination with 1 mM ectoine, and cell free BAL obtained from animals which were treated with CNP alone plus 1 mM ectoine in PBS *in vitro*. Cell viability was tested by trypan blue (Sigma Aldrich) exclusion test. BMDCs were isolated and stained for flow cytometry for expression of CCR7.

### Statistical analyses

Statistical calculations were performed using IBM SPSS statistics 22. Significant values were calculated either by ANOVA analyses with Tukey’s HSD post hoc testing or by comparison of individual groups by Mann–Whitney *U*-test. Mean values with standard errors are given. Power calculations for the design of animal experiments were performed using G*Power version 3.1.5 (University of Kiel, Germany). Graphs display means and standard errors.

## Additional file

Additional file 1:
**Figure S1.** Primary physical characteristics of CNP determined as suspension in H_2_O. A. Transmission elecron micrograph. B. Agglomerate size distribution. **Table S1.** Physical charcteristics of CNP suspension in PBS.
