# Supplementary material for: Preventing carbon nanoparticle-induced lung inflammation reduces antigen-specific sensitization and subsequent allergic reactions in a mouse model
Source: Part Fibre Toxicol. 2015 Jul 4;12:20. doi: 10.1186/s12989-015-0093-5 (PMC4491258; doi:10.1186/s12989-015-0093-5)
Supplement: Additional file 1: — Figure S1. Primary physical characteristics of CNP determined as suspension in H2O. A. Transmission elecron micrograph. B. Agglomerate size distribution. Table S1. Physical charcteristics of CNP suspension in PBS. [file 12989_2015_93_MOESM1_ESM.pdf]

Preventing carbon nanoparticle induced lung inflammation reduces antigen-specific sensitization and subsequent allergic reactions in a mouse model

Matthias Kroker, Ulrich Sydlik, Andrea Autengruber, Christian Cavelius, Heike Weighardt, Annette Kraegeloh, Klaus Unfried

Physical analysis of carbon nanoparticle suspensions
Method: Particles were suspended in PBS [1 mg/ml] by ultrasound desintegration. Suspensions were subjected to transmission electron microscopy (JEM-2100 LaB6, JEOL, Tokyo, Japan) and dynamic light scattering (Zetasizer, Malvern Instruments, Malvern, UK). Analyses were performed in triplicate. When applicable, means and standard deviations were calculated.

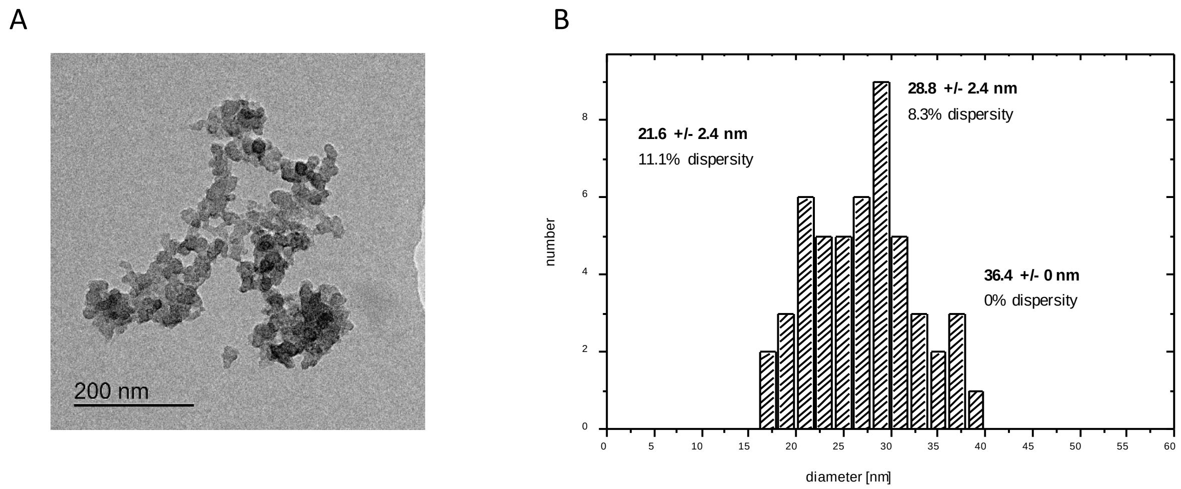

Figure S1
Primary physical characteristics of CNP determined as suspension in H_2O. A. Transmission elecron micrograph. B. Agglomerate size distribution.

Table S1: Physical charcteristics of CNP suspension in PBS.

**zeta potential** [mV]	**mobility** [µmcm/Vs]	**conductivity** [mS/cm]	**size** [nm] peak1 [%]	**size** [nm] peak2 [%]	**size** [nm] peak3 [%]
-22.4	-1.755	-16.56	887 (±197)<br>88.8	118 (±126)<br>10.5	1798 (±3114.8)<br>0.7
